# Genetic diversity associated with natural rubber quality in elite genotypes of the rubber tree

**DOI:** 10.1038/s41598-020-80110-w

**Published:** 2021-01-13

**Authors:** Isabela de Castro Sant’Anna, Ligia Regina Lima Gouvêa, Maria Alice Martins, Erivaldo José Scaloppi Junior, Rogério Soares de Freitas, Paulo de Souza Gonçalves

**Affiliations:** 1 Center of Rubber Tree and Agroforestry Systems, Agronomic Institute (IAC), Votuporanga, Brazil; 2 Agronomic Institute, Seringueira Program, Campinas, Brazil; 3 Embrapa Instrumentation, Nanotechnology National Laboratory for Agriculture (LNNA), São Carlos, Brazil

**Keywords:** Genetics, Plant sciences

## Abstract

The objective of this study was to evaluate the genetic variability of natural rubber latex traits among 44 elite genotypes of the rubber tree [*Hevea brasiliensis* (Willd. ex Adr. de Juss.) Müell. Arg.]. Multivariate analysis and machine learning techniques were used, targeting the selection of parents that demonstrate superior characters. We analyzed traits related to technological or physicochemical properties of natural rubber latex, such as Wallace plasticity (P_0_), the plasticity retention index [PRI (%)], Mooney viscosity (V_R_), ash percentage (Ash), acetone extract percentage (AE), and nitrogen percentage (N), to study genetic diversity. Multivariate [unweighted pair group method with arithmetic means (UPGMA) and Tocher)] and machine learning techniques [K-means and Kohonen’s self-organizing maps (SOMs)] were employed. The genotypes showed high genetic variability for some of the evaluated traits. The traits PRI, Ash, and P_O_ contributed the most to genetic diversity. The genotypes were classified into six clusters by the UPGMA method, and the results were consistent with the Tocher, K-means and SOM results. PRI can be used to improve the industrial potential of clones. The clones IAC 418 and PB 326 were the most divergent, followed by IAC 404 and IAC 56. These genotypes and others from the IAC 500 and 400 series could be used to start a breeding program. These combinations offer greater heterotic potential than the others, which can be used to improve components of rubber latex quality. Thus, it is important to consider the quality of rubber latex in the early stage of breeding programs.

## Introduction

The rubber tree [*Hevea brasiliensis (*Willd. ex Adr. de Juss.) Müell. Arg.] is native to the Amazon region. However, since the introduction of rubber tree materials by Henry Wickham in 1876, Southeast Asia has become the major producer of natural rubber^1^. Thus, rubber production worldwide is derived from a small number of collected seeds with a narrow genetic base^2^.

Worldwide production has increased in the last 30 years as a result of an increase in the rubber agricultural planted area and a large increase in productivity, to which genetic improvement remains a key contributor^3^. The success of crop improvement depends on the extent of genetic diversity available in germplasms as well as information on important traits. Recently, many studies have been conducted in germplasm banks in countries such as Vietnam^3^, Malaysia^4,5^, Nigeria^6^, Sri Lanka^7^, Brazil^8–10^ and India^11^. Genetic variability studies are crucial for the selection of parents for hybridization^12^. However, breeding program efforts have focused mainly on advanced varieties with improved production, vigor, disease resistance and the shortening of the crop breeding cycle to release new clones^13^, and information on selection for the quality of the rubber generated is lacking. Such information is crucial and may determine the applications of this natural polymer^14^.

Natural rubber is a biopolymer consisting of (C5H8)n isoprene units and other cellular components, such as proteins, minerals, carbohydrates and lipids. These components confer rubber compounds elasticity, resilience and toughness. Natural rubber is an essential raw material for more than 50,000 products, such as medical devices, surgical gloves, aircraft and car tires, clothes, and toys, and cannot be replaced by synthetic rubber in many cases^15^. Differences in the composition of rubber components affect the predominance of certain properties and lead to different selling prices.

In Brazil, most studies on this topic are aimed at determining whether the rubber produced nationally meets the standards required by Brazilian legislation^16–20^. In Colombia, some authors have studied the components of rubber to evaluate the quality of rubber genotypes and found variability among clones in these components^21,22^. It is important to consider such information in the early stages of a breeding program.

Therefore, the present work was aimed at evaluating the diversity among elite genotypes in traits related to technological or physicochemical properties of the rubber tree latex. The findings may inform genetic breeding programs regarding the crossing of different rubber genotypes. In particular, it might be possible improve specific traits of genotypes related to the industrial sectors most suitable for their application.

## Results

### Analysis of genetic diversity

The Tocher optimization method grouped the 44 rubber tree genotypes into six clusters (Table 1). Twenty-five genotypes were grouped in cluster I, which was composed of Asiatic genotypes (PB 311, PB 312, PB 314, PB 324, RRIM 600, RRIM 713, and RRIM 901) and Brazilian genotypes (members of the IAC 300 series, IAC 40, IAC 56, and all members of the IAC 400 series except IAC 418). Cluster II was composed of six Asiatic genotypes (PM 10, RRIM 938, PB 291, PC 119, PB 350, and RRIM 937) and IAC 507. Cluster III comprised eight genotypes from the IAC 500 series. Cluster IV comprised PB 355, cluster V comprised GT1 and PB 326, and cluster VI comprised IAC 418.

**Table 1:** Classification of 44 rubber tree genotypes into different clusters on the basis of divergence.

Cluster ID	Number of genotypes	Genotypes
1	18	IAC406 IAC410 IAC411 IAC417 IAC412 IAC405 IAC409 IAC404 IAC300 IAC401 IAC301 IAC40 IAC56 IAC403 IAC400 AC302 IAC407 RRIM600
2	13	GT1 PB291 PB311 PB312 PB314 PB355 PB350 PC119 PM10 RRIM713 RRIM901 RRIM937 RRIM938
3	8	IAC500 IAC501 IAC502 IAC503 IAC505 IAC506 IAC507 IAC511 IAC512
4	1	PB355
5	1	PB326
6	1	IAC418

**Figure 1:**
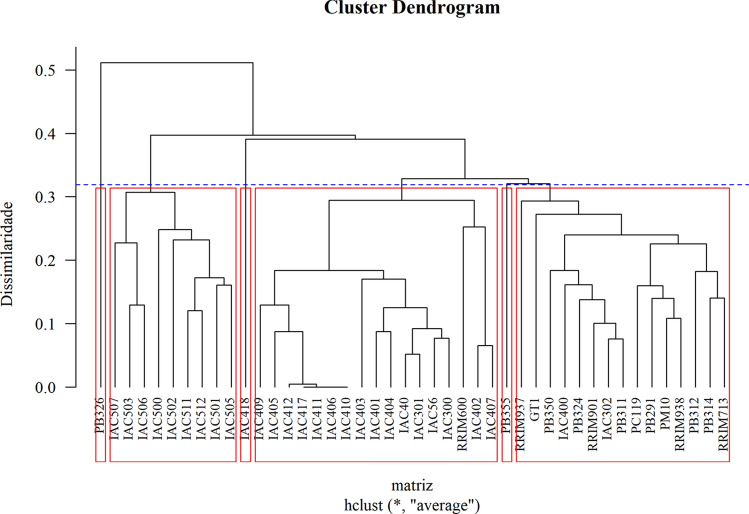
UPGMA cluster analysis of Euclidean distances based on data on the technological properties of natural rubber latex, used in the evaluation of the 44 rubber tree genotypes. The red line highlight the groups identified by K = 1.25; the cophenetic correlation was 0.77. From left to right: group I: PB 326; group II: IAC 500 series; group III: IAC 418; group IV: IAC 400 series, IAC 300, IAC 301, IAC 40, IAC 56 and RRIM600; group V: PB 355; group VI: Asiatic genotypes, IAC 400 and IAC 302. The percentage distance between cultivars is presented on the Y axis, and the 44 genotypes are presented on the X axis.

The results of the unweighted pair group method with arithmetic means (UPGMA) agreed with the results of the Tocher method, with small differences (Fig. 1). The cophenetic correlation was r = 0.77, indicating that the dendrogram clustering accurately depicted the estimated genetic distances among the genotypes. This value was significant at the 1% probability level by the Mantel test, based on resampling 1000 times. A total of six groups were identified at K = 1.25, as suggested by Milligan and Cooper^23^. The IAC 418 and PB 326 genotypes were highly distinct from each other based on Euclidean distance (D = 0.717). The lowest genetic dissimilarity among genotypes was found among IAC 406, IAC 410 and IAC 411.

**Figure 2:**
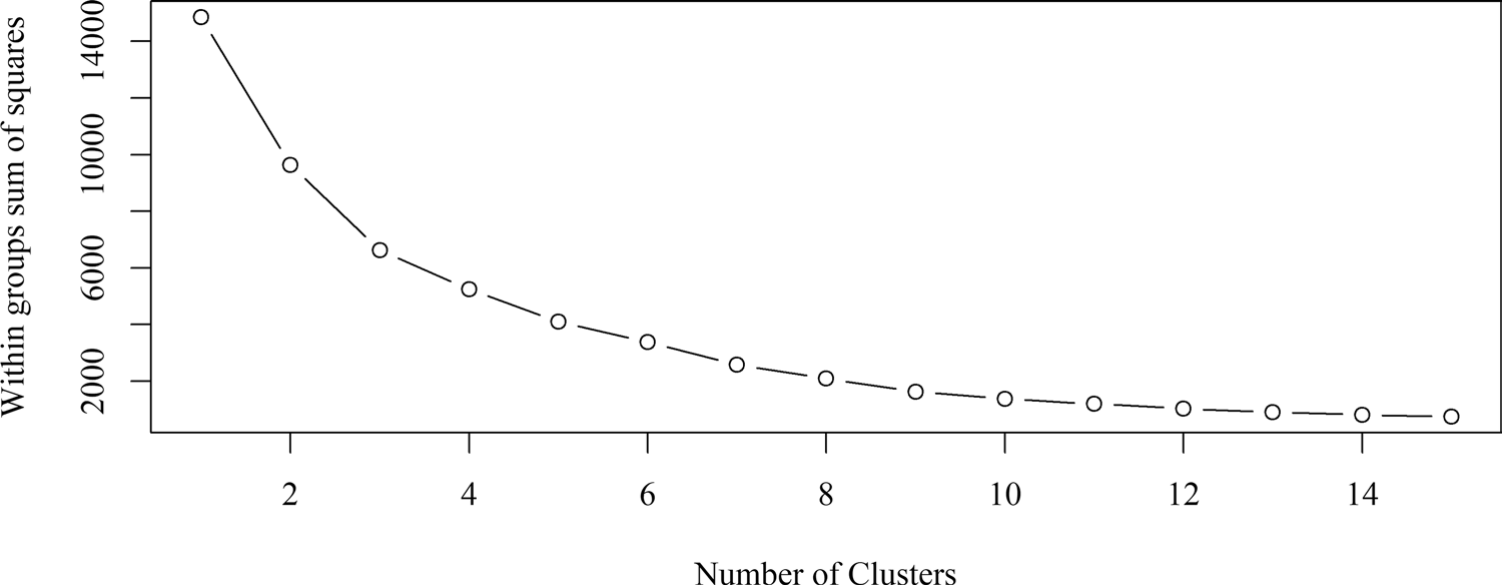
Sum of squares graph plot for different numbers of clusters. The best ‘k’ is chosen at the point where the marginal gain sharply decreases, yielding an angle in the graph (the “elbow” criterion); in our case, the value is six.

**Figure 3:**
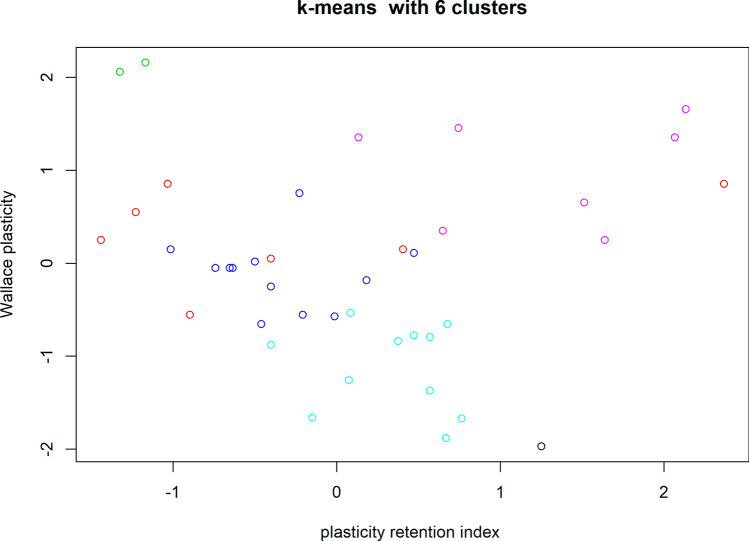
K-means analysis of analysis of data on technological properties of natural rubber, used in the evaluation of the 44 rubber tree genotypes. Axes represent the first two components (Comp 1 and 2). Each dot represents an individual. The colors represent different clusters identified by the analysis. From left to right: cluster V is shown in green and comprises IAC 402 and IAC 407, cluster III is shown in red and comprises Asiatic genotypes (PB 291, PB 314, PB 355, PC 119, PM 10, RRIM 713, RRIM 937, and RRIM 938), cluster I is shown in blue and comprises IAC genotypes except members of the IAC 500 series. Cluster VI is shown in aqua and it is formed by the other genotypes from Asia (PB 311, PB 312, PB 324, PB 350, RRIM 901, GT1), cluster IV is shown in black and includes only PB 326, and cluster II is composed of the IAC 500 series.

The number of clusters was confirmed iteratively by the elbow method^24^ (Fig. 2) to be six in accordance with the number of groups provided by the Tocher method and UPGMA. The 44 genotypes were divided into six distinct groups by K-means cluster analysis (Fig. 3); the group membership of each genotype was identified by one of six colors. Cluster I (blue) was composed of all Agronomic Institute (IAC) genotypes except the 500 (pink) series; cluster II comprised IAC 500 series genotypes. Cluster III (red) comprised Asiatic genotypes (PB 291, PB 314, PB 355, PC 119, PM 10, RRIM 713, RRIM 937, and RRIM 938). Cluster IV (black) comprised PB 326, and cluster V (green) comprised IAC 402 and IAC 407. The last cluster, cluster VI (aqua), was formed by the remaining genotypes from Asia. With this partitioning, the variation among groups corresponded to 65.39% of the total variation, whereas the variation within groups represented only 34.61%.

**Figure 4:**
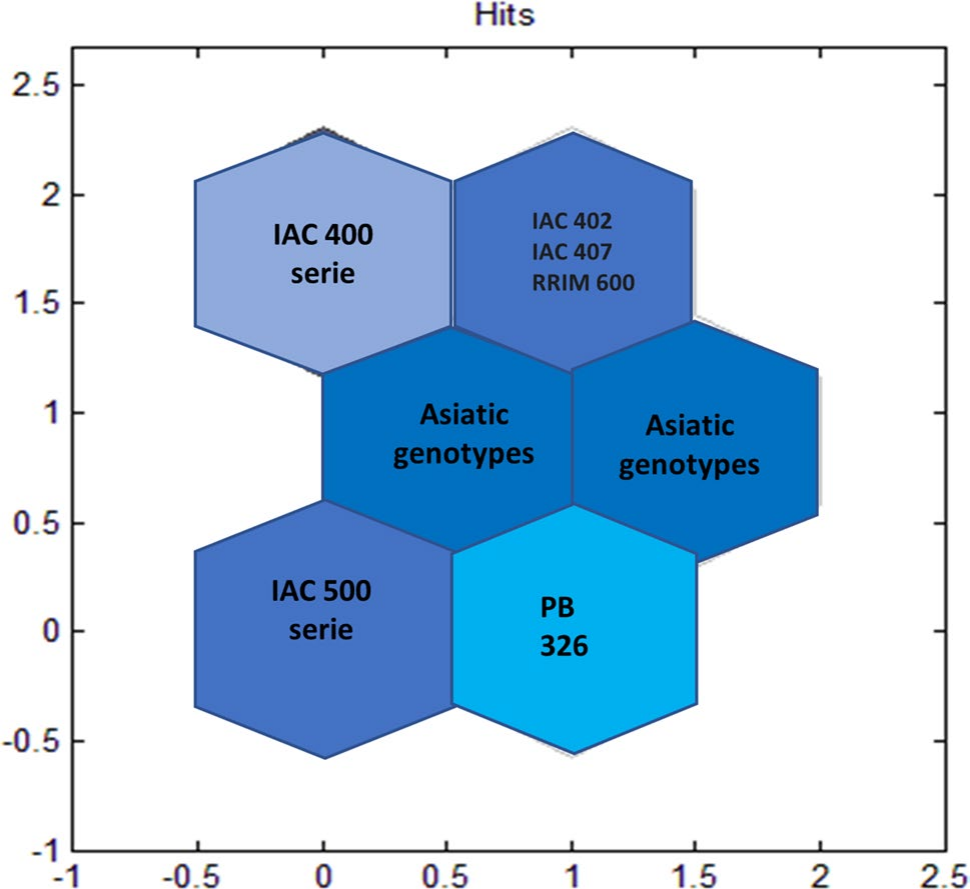
For self-organizing maps (SOMs), six clusters were formed. From left to right and from bottom to top: Cluster I comprises the IAC 500 series; cluster II comprises PB 326; cluster III comprises IAC 507 and Asiatic genotypes (PB 291, PB 314, PB 355, PC 119, PM 10, RRIM 713, RRIM 937, and RRIM 938); cluster IV comprises some Asiatic genotypes (PB 311, PB 312, PB 324, PB 350, RRIM 901, GT1), IAC 302 and IAC 400; cluster V comprises the IAC 400 series (IAC 401, IAC403, IAC 404, IAC 405, IAC 406, IAC 409, IAC 410, IAC 411, IAC 412, IAC 417, and IAC 418); and cluster VI comprises the genotypes IAC 402, IAC 407 and RRIM 600.

**Figure 5:**
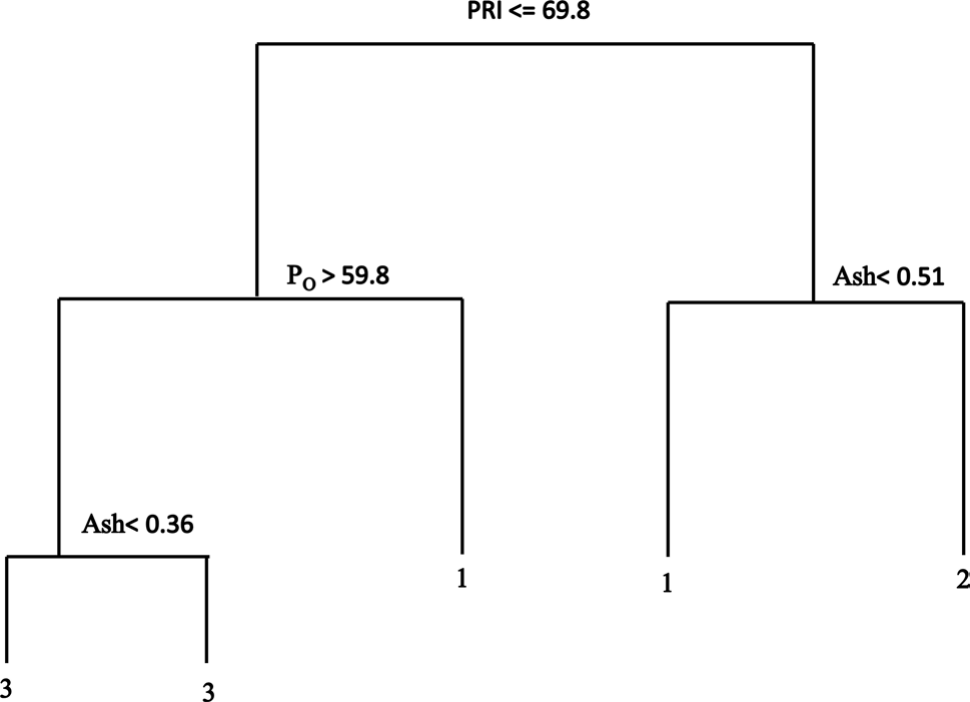
Decision trees for the natural rubber compounds of rubber tree. The root of the tree is divided into two nodes according to PRI < 69.8% (left branch) and PRI ≥ 69.8% (right branch). The right branch is divided according to Ash, yielding Node 3 [Ash < 0.51% (cluster 1)] and Node 4 [ASH > 0.51% (cluster 2)]. The left branch is divided according to Po [Node 5 Po < 0.36 and Node 6 Po ≥ 0.36]. The classification used only three clusters, the first one consisting of all IAC genotypes except the IAC 500 series, the second comprising the IAC 500 series and the third one comprising the Asiatic genotypes.

For self-organizing maps (SOMs; Fig. 5), it was iteratively shown that the best network architecture involved three columns and four rows, hextop as the topology, Euclidean distance, 3000 epochs and a spread of six neurons. The SOM results were in accordance with the k-means results. Six clusters were formed: Cluster I comprised the IAC 500 series, cluster II comprised PB 326, cluster III comprised IAC 507 and Asiatic clones (PB 291, PB 314, PB 355, PC 119, PM 10, RRIM 713, RRIM 937, and RRIM 938), and cluster IV comprised some Asiatic genotypes (PB 311, PB 312, PB 324, PB 350, RRIM 901, and GT1), IAC 302 and IAC 400. In cluster V were members of the IAC 400 series (IAC 401, IAC 403, IAC 404, IAC 405, IAC 406, IAC 409, IAC 410, IAC 411, IAC 412, IAC 417, and IAC 418). In cluster VI were IAC 402, IAC 407 and RRIM 600 (Fig. 4).

### Variable measurement importance

The principal component analysis (PCA) of natural rubber components (Table 2) showed that the first three principal components accounted for 77.39% of the total variation. Among the variables, the plasticity retention index (PRI) contributed the most to the estimated genetic divergence among the 44 genotypes. The acetone extract percentage (AE) was the next largest contributor among the last three main components; the remainder contributed little to the genotypic diversity, being redundant or invariant.

**Table 2:** Principal components and the explained variance in the genetic diversity of traits related to technological properties of natural rubber latex, used in the evaluation of the 44 rubber tree genotypes. The first three principal components explained 80% of the variation among genotypes.

Component	PC 1	PC 2	PC 3	PC 4	PC 5	PC 6	PC 7
Eigenvalue	3.05	1.51	0.97	0.64	0.41	0.263	0.15
Cumulative variance	43.61	65.23	79.09	88.23	94.09	97.84	100

In the decision tree (Fig. 5) for the natural rubber compounds, the most important variable was located at the root of the tree and was divided into two nodes according to PRI < 69.8% (left branch) or PRI ≥ 69.8% (right branch). The right branch was divided by ash percentage (Ash) [Node 3: Ash < 0.51% (cluster 1); Node 4: Ash > 0.51% (cluster 2)]. The left branch was divided by Wallace plasticity (Po) subgroup (Node 5: Po < 0.36 and Node 6: Po ≥ 0.36). In this analysis, it was not possible to obtain six groups because clusters with fewer than two genotypes were not accepted. Thus, the classification used only three clusters, with the first comprising all Brazilian clones except the 500 series, the second comprising the IAC 500 series and the third comprising Asiatic clones.

## Discussion

### Genetic diversity

The genetic improvement of important traits in plant breeding depends upon the genetic diversity available within the species of interest. Here, the genetic diversity of 44 clones was studied using traits related to the technological properties of natural rubber latex, resulting in the identification of distinct groups for the IAC series and for the Asiatic clones. The genotypes with high levels of genetic variation found in this study are beneficial resources for breeding programs aimed at improving the quality of natural rubber. The number of defined groups showed agreement between the traditional techniques applied for assessing genetic diversity (UPGMA and Tocher) and both the unsupervised learning technique of k-means analysis and the evaluation of SOMs. It was important to demonstrate the reliability of the results obtained here.

There was a clear relationship between the cluster allocation and genotype origin, as defined according to the types of crosses realized in the breeding programs that gave rise to the materials (Table 3 and Fig. 1). According to the UPGMA results, clusters II, III and IV were formed from Brazilian genotypes included in the rubber tree breeding program at the Agronomic Institute that had been selected in different breeding cycles. Cluster II corresponded to the IAC 500 series^25^, cluster III corresponded to genotype IAC 418, and cluster IV corresponded to the IAC 300 series^26^ and IAC 400 series^27^. Clusters I and V comprised PB 326 and PB 355, respectively. Cluster VI was composed of Asiatic genotypes. As shown in Fig. 1, in clusters I and V, there was a unique genotype from the Prang Besar (PB) plantations. In cluster II, the genotypes from the IAC 500 series were all illegitimate; that is, they were the result of open pollination in female parents selected by different institutions. Cluster VI was composed of genotypes selected in Malaysian breeding programs at the Rubber Research Institute of Malaysia (RRIM) and from the PB plantations; these genotypes are descendants of Wickham clones^28^. In cluster III, the IAC 300 series members were the result of crosses performed via controlled pollination between Malaysian and Indonesian clones, wherein the Malaysian parents were selected from RRIM, and the Indonesian parents were selected from the experimental station of Algemene Vereniging Rubber Planters Oostkust Sumatra (AVROS). The genotypes from the IAC 400 series were obtained through controlled pollination and open pollination. The clones used as parents for the IAC 400 series of genotypes and the Asian clones came from the genitors RRIM 600, GT 711, PB 86, Tjir 1, and PB 235, among others. In complex scenarios such as this in which the genetic similarity between genotypes can differ (siblings, half siblings, parents and grandparents), the SOM method allows the visualization of patterns of similarity and data classification based on the distances between genotypes^29^. This method is efficient, as noted by^30–32^. Thus, the agreement between most of the applied techniques, especially between the K-means and SOM methods, suggested that the Asiatic clones were best represented as two clusters, as were the IAC 400 series. The genotypes of Asiatic clones with RRIM 600 as the genitor were more closely related to clones IAC 402, IAC 407 and RRIM 600 than to the other Asiatic clones with a different genitor. These results agree with those found by Amorim et al.^33^, who evaluated the genetic divergence of sunflower and observed that the genotypes used in the Brazilian and Argentine breeding programs separated into distinct groups. However, other authors have not found a relationship between the formation of clusters and genotypic origin. For example, Vog et al.^34^ detected no difference between groups formed among 17 sunflower cultivars from different breeding programs according to Argentine versus Brazilian origin. Carmo et al.^35^ studied fava beans and noticed that the cultivar groups that were formed corresponded to different countries.

**Table 3:** Genealogies and origin of 44 rubber tree genotypes used in the study of genetic divergence related to natural rubber quality. Some of the genotypes are pedigree genotypes from Asia; most are from the IAC breeding program in Brazil. Both types were evaluated in Votuporanga, SP.

Genotype	Genealogy	Origin
GT 1	Primary clone	Indonesia
IAC 40	RRIM 608 (Tjir 33 × Tjir 1) × AVROS 1279 (AVROS 156 × AVROS 374)	Brazil
IAC 56	RRIM 608 (Tjir 33 × Tjir 1) × Fx 3810	Brazil
IAC 300	RRIM 605 (Tjir 1 × PB 49) × AVROS 353 (AVROS 164 × AVROS 160)	Brazil
IAC 301	RRIM 501 (Pil A 44 × Lun N) × AVROS 1518 (AVROS 214 × AVROS 317)	Brazil
IAC 302	RRIM 501 (Pil A 44 × Lun N) × AVROS 353 (AVROS 164 × AVROS 160)	Brazil
IAC 400	GT 711 × RRIM 600 (Tjir 1 × PB 86)	Brazil
IAC 401	RRIM 600 (Tjir 1 × PB 86) ill.^(2)^	Brazil
IAC 402	GT 711 ill.^(2)^	Brazil
IAC 403	GT 711 ill.^(2)^	Brazil
IAC 404	PB 5/63 (PB 56 × PB 24) × AVROS 363	Brazil
IAC 405	Tjir 1 × RRIM 623 (PB 49 × Pil B 84)	Brazil
IAC 406	IAN 873 (PB 86 × FA 1717) × RRIM 600 (Tjir 1 × PB 86)	Brazil
IAC 407	RRIM 600 (Tjir 1 × PB 86) ill.^(2)^	Brazil
IAC 409	Fx 2784 (F 4542^(1)^ × AVROS 363) ill.^(2)^	Brazil
IAC 410	PB 86 × PB 235 [PB 5/51 (PB 56 × PB 24) × PB S/78 (PB 49 × PB 25)]	Brazil
IAC 411	GT 711 ill.^(2)^	Brazil
IAC 412	IAN 873 (PB 86 × FA 1717) × GT 711	Brazil
IAC 417	RRIM 600 (Tjir 1 × PB 86) ill.^(2)^	Brazil
IAC 418	RRIM 600 (Tjir 1 × PB 86) ill.^(2)^	Brazil
IAC 500	RRIM 600 (Tjir 1 × PB 86) ill.^(2)^	Brazil
IAC 501	RRIM 526 (Pil B 58 × Pil D 65) ill.^(2)^	Brazil
IAC 502	IAC 41 [RRIM608 (Tjir 33 × Tjir 1) × AVROS 1279 (AVROS 156 × AVROS 374)] ill.^(2)^	Brazil
IAC 503	Fx 3899 (F 4542 ^(1)^ × AVROS 363) ill.^(2)^	Brazil
IAC 505	IAN 873 (PB 86 × FA 1717) ill.^(2)^	Brazil
IAC 506	AVROS 1513 ill	Brazil
IAC 507	IAC 90 [RRIM 507 (Pil B 84 × Pil A 44) × Fx 25(F 351 × AVROS 49)] ill	Brazil
IAC 511	IAC 15 [RRIM 504 (Pil A 44 × Lun N) × RRIM 600(Tjir 1 × PB 86)] ill.^(2)^	Brazil
IAC 512	Fx 25 (F 351 × AVROS 49) ill.^(2)^	Brazil
PB 291	Unknown*	Malaysia
PB 311	RRIM 600 (Tjir 1 × PB 86) × PB 235 [PB 5/51 (PB 56 × PB 24) × PB S/78 (PB 49 × PB 25)]	Malaysia
PB 312	RRIM 600 (Tjir 1 × PB 86) × PB 235 [PB 5/51 (PB 56 × PB 24) × PB S/78 (PB 49 × PB 25)]	Malaysia
PB 314	RRIM 600 (Tjir 1 × PB 86) × PB 235 [PB 5/51 (PB 56 × PB 24) × PB S/78 (PB 49 × PB 25)]	Malaysia
PB 324	RRIM 600 (Tjir 1 × PB 86) × PB 235 [PB 5/51 (PB 56 × PB 24) × PB S/78 (PB 49 × PB 25)]	Malaysia
PB 326	RRIM 600 (Tjir 1 × PB 86) × PB 235 [PB 5/51 (PB 56 × PB 24) × PB S/78 (PB 49 × PB 25)]	Malaysia
PB 350	RRIM 600 (Tjir 1 × PB 86) × PB 235 [PB 5/51 (PB 56 × PB 24) × PB S/78 (PB 49 × PB 25)]	Malaysia
PB 355	PB 235 [PB 5/51 (PB 56 × PB 24) × PB S/78 (PB 49 × PB 25)] × PR 107	Malaysia
PC 119	GT 1 × RRIM 703 [RRIM 600 (Tjir 1 × PB 86) × RRIM 500 (Pil B 84 × Pil A 44)]	
PM 10	Unknown	Malaysia
RRIM 600	Tjir 1 × PB 86	Malaysia
RRIM 713	RRIM 605 (Tjir 1 × PB 49) × RRIM 71	Malaysia
RRIM 901	PB 5/51 (PB 56 × PB 24) × RRIM 600 (Tjir 1 × PB 86)	Malaysia
RRIM 937	PB 5/51 (PB 56 × PB 24) × RRIM 703 [RRIM 600 (Tjir 1 × PB 86) × RRIM 500 (Pil B 84 × Pil A 44)]	Malaysia
RRIM 938	PB 5/51 (PB 56 × PB 24) × RRIM 703 [RRIM 600 (Tjir 1 × PB 86) × RRIM 500 (Pil B 84 × Pil A 44)]	Malaysia

*Hevea benthamiana* genotype*.* ^(2)^ill., illegitimate (genotype obtained from an open pollination matrix plant). ^(3)^Amazonian genotypes (*F* Ford; *FA* Ford Acre, *Fx* Ford crossing, *IAN* Instituto Agronômico do Norte; *RO* Rondônia); São Paulo genotypes (*IAC* Instituto Agronômico), Indonesian genotypes (*AVROS* Algemene Vereniging Rubberplanters Oostkust Sumatra, *GT* Godang Tapen, *PR* Proefstation voor rubber, *Tjir* Tjirandji); Malaysian genotypes (*Lun* Lunderston, *PB* Prang Besar, *Pil* Pilmoor, *RRIM* Rubber Research Institute of Malaysia).

The identification of the genetic relationships and divergence among genetic resources is useful for the selection of parental genotypes in breeding programs^12^. The current study was carried out to establish the genetic diversity and relationships among rubber tree genotypes to identify appropriate parents for hybridization. The use of parental genotypes with the greatest possible divergence is important to maximize heterosis in hybrids and increase the genetic base^12^. The most divergent pair of genotypes was IAC 418 and PB 326 (D = 0.717), followed by IAC 404 and IAC 56 (D = 0.648). According to the SOM results that organized genetic diversity, the genotypes from the IAC 400 and IAC 500 series could also be crossed.

The least similar pairs of genotypes were those formed among IAC 406, IAC 410, IAC 412, and IAC 417; these genotypes were are similar to one another in all the traits examined in this study. In general, the shortest distances were found within groups and the largest between groups. The most appropriate strategy would be to prioritize crossings between individuals from different groups, as suggested by Cruz, et al.^12^. Although the studied genotypes consisted exclusively of high-production genotypes in Brazil, genetic diversity within the breeding program has been maintained for the studied traits. Different plant breeding methods show different impacts on plant genetic diversity, and the kind of crossing applied in each series likely helps maintain genetic diversity.

According to Fu^36^, no consensus has been reached regarding the overall impact of modern plant breeding on crop genetic diversity. The author emphasized that the temporal patterns of crop genetic diversity are largely inconsistent with our perception that modern plant breeding reduces crop genetic diversity. For example, Wouw et al.^37^ performed a meta-analysis of 44 published diversity assessments and indicated that a gradual narrowing of the genetic base of the varieties released by breeders could not be observed. A similar result was found here: the grouping promoted by Tocher’s method, based on Euclidean genetic distances, resulted in six mutually exclusive groups (Table 1). The grouping pattern showed that 56.81% of the genotypes belonged to cluster I, which was composed mainly of IAC 300 and 400 series and Asiatic genotypes and that 15.90% of the genotypes belonged to cluster II, which was composed of Asiatic clones and IAC 507. Another 18.18% of the genotypes belonged to cluster III and were members of the 1AC 500 series, and each of the single genotypes in clusters IV, V and VI represented 2.2% of the total.

### Variable measurement

The present study investigated six traits associated with the technological properties of natural rubber latex to reveal the genetic diversity among 44 elite genotypes that are among the most commonly used genotypes in Brazil. The traits that contributed most to the observed genetic divergence were PRI, Ash, and P_O_, as suggested by the decision tree analysis. The PRI is used to evaluate resistance to thermoxidative degradation in natural rubber^19^. According to the Brazilian standard, for the raw material to be considered of good quality, it must have a PRI value equal to or higher than 50%^38^. The studied genotypes in the present study showed an average PRI value of 68.50 ± 7.61%. Those in cluster III exhibited PRI values equal to or greater than 69.8%, whereas those in cluster IV showed values below 69.8%.

The trait Po provides an estimate of the length of the polymeric chain and the state of degradation of the raw material^17^. The studied genotypes exhibited an average P_O_ value of 57.69 ± 9.9%. The genotype with the highest mean value was IAC 402 (Po = 90 ± 9%) and that with the lowest was PB 326 (Po = 38.9 ± 9%). All of the genotypes studied exhibited an average Po value higher than the minimum established by the standard (Po = 30)^39^, indicating that they produce rubber with long polymer chains. Mooney viscosity (V_R_) indicates the resistance of natural rubber to a rotor operating at a constant speed at ML (1 + 4) 100°C^17^. The average value of this trait across the genotypes was approximately V_R_ = 99.46, with the highest value found for RRIM 713 (V_R_ = 118) and the lowest for PB 326 (V_R_ = 65). The IAC 500 series presented an average of V_R_ = 84. Considering the diversity identified in this study, much can be done to explore this genetic variability depending on the interests of the industry, starting with the direction of specific crosses.

The Ash determination test reduces rubber to only those inorganic components that do not decompose at a temperature of approximately 600 °C, with all substances of an organic nature being destroyed at this temperature. In addition to reducing the dynamic properties of the vulcanized material, an excess Ash content can negatively influence its aging properties. All genotypes showed a percentage of Ash within the value stipulated by the standard, with average variation of 0.50 ± 0.18%^18,40^.

In general, the studied traits are very important for the industrial application of the evaluated genotypes. For example, the acetone extract (AE) content test consists of the extraction of substances that are soluble in acetone, among which lipids are the main components. Studies show that the AE content can vary from 2 to 5% in dry rubber; the Brazilian standard establishes a maximum value of 3.5%^18,41^. The evaluated genotypes showed an average value of 3.31 ± 0.8%. Among the genotypes, those in clusters I and II presented values below 3.23%.

The trait nitrogen content (N) is indicative of the contents of proteins, amino acids and nitrogenous bases that are present in latex and remain in natural rubber after coagulation^42,43^. According to Brazilian legislation, to be considered of good quality, natural rubber must present an N value between 0.2 and 0.6%, and the standard establishes 0.6% as the maximum value^44^. The results showed that the studied genotypes presented an average value of 0.496 ± 0.05%, which is in accordance with the current standard.

Although the traits AE, N, and V_R_ had little importance in differentiating the studied genotypes, the determination of these parameters is extremely important for industries, as they are indicators of natural rubber behavior during processing and the quality of the feedstock. In addition, other important relationships might be found with other data sets from different locations.

## Material and methods

Natural rubber latex from 44 genotypes was used in this study; the genealogies of these genotypes are described in Table 3. The performance of these genotypes has been evaluated at the Center of Rubber Tree and Agroforestry Systems, IAC, Votuporanga (São Paulo state, Brazil), at 20°20′S, 49°58′W and an altitude of 510 m. The soil is characterized as Arenic Hapludult^45^. The Asiatic clones were introduced into Brazil by Embrapa (Brazilian Agricultural Research Corporation) at the end of the last century. Most are pedigree genotypes originating from Asiatic breeding programs. Together with the Brazilian genotypes from the IAC breeding program, the Asiatic genotypes were evaluated for growth and yield. Panel tapping was initiated when the trees were 7 years old and was followed by the half-spiral-cut tapping system, with tapping conducted every 4 days and latex production stimulated with 2.5% ethephon eight times per year. After 3 years of tapping, latex samples from 30 trees per genotype were collected and sent to Nanotechnology National Laboratory for Agriculture (LNNA) at Embrapa Instrumentação for natural rubber analysis.

## Methods

### Compound assessment

The technological properties of the natural rubber were evaluated by Embrapa Agricultural Instrumentation, São Carlos, Sao Paulo state, following standard procedures described by the Brazilian Association of Technical Standards (ABNT). Assays for the following parameters were performed: Wallace plasticity (Po)^39^, the plasticity retention index (PRI)^38^, Mooney viscosity (V_R_)^46^, ash percentage (Ash)^47^, acetone extract percentage (AE)^41^, and nitrogen percentage (N)^44^. The tests were performed in duplicate except for Po and PRI, which were analyzed in quintuplicate. Brazilian standards are identical in technical content, design and writing to the ISO standards for natural rubber that were developed by the Technical Committee Rubber and Rubber Products (ISO/TC 45) according to ISO/IEC Guide 21-1:2005^48^. The RRIM 600 clone was analyzed for validation purposes as it is the most planted clone under the cultivation conditions of rubber plantations.

### Multivariate analysis

Cluster analysis was performed on standardized morphological data based on the average Euclidian distance coefficient and UPGMA. The cophenetic correlation was estimated, and its significance was tested by the Mantel test based on resampling 1000 times. In addition, the Tocher optimization method was applied.

The K-means algorithm is a simple unsupervised machine-learning algorithm that groups data into a specified number (k) of clusters. Because the user must specify in advance what k to choose, the algorithm is somewhat naive—it assigns all members to k clusters even if the k value is not the appropriate k for the dataset. Therefore, the number of clusters was confirmed iteratively by the elbow method^24^. The K-means algorithm cluster analysis was performed using R^49^ and the Genes program^50^.

Kohonen’s self-organizing maps (SOMs) were used to evaluate the organization of diversity in the software MATLAB Version 7.10.0^51^ and GENES^50^. Different network architectures were tested by varying the number of rows (1 to 5) and columns (1 to 5). The defined topology was hextop (i.e., with a hexagonal neighborhood), and the distance used to configure the artificial neural networks was the Euclidean distance.

### Variable importance measures

PCA^52^ and decision trees^53^ were used to determine the contribution of traits to the diversity of genotypes.

## Conclusions

The genetic diversity of rubber tree genotypes was analyzed, with genotypes clustered into distinct groups. Among the evaluated traits, those that contributed the most to genetic divergence were PRI, Ash, and P_O_. The greatest divergence was observed between IAC 418 and PB326, followed by IAC 404 and IAC 56. These genotypes and others from the IAC 500 and 400 series could be used to start a breeding program. The findings indicate a greater heterotic potential of these combinations than of others that can be used to improve components of natural rubber quality. It is important to include the assessment of the quality of rubber latex in the early stage of breeding programs.
